# Global Gene Expression Alterations as a Crucial Constituent of Human Cell Response to Low Doses of Ionizing Radiation Exposure

**DOI:** 10.3390/ijms17010055

**Published:** 2015-12-31

**Authors:** Mykyta Sokolov, Ronald Neumann

**Affiliations:** Nuclear Medicine Division, Department of Radiology and Imaging Sciences, Clinical Center, National Institutes of Health, 9000 Rockville Pike, Bethesda, MD 20892, USA; rneumann@mail.nih.gov

**Keywords:** human cell, ionizing radiation, low dose, gene expression, DNA microarray

## Abstract

Exposure to ionizing radiation (IR) is inevitable to humans in real-life scenarios; the hazards of IR primarily stem from its mutagenic, carcinogenic, and cell killing ability. For many decades, extensive research has been conducted on the human cell responses to IR delivered at a low dose/low dose (LD) rate. These studies have shown that the molecular-, cellular-, and tissue-level responses are different after low doses of IR (LDIR) compared to those observed after a short-term high-dose IR exposure (HDIR). With the advent of high-throughput technologies in the late 1990s, such as DNA microarrays, changes in gene expression have also been found to be ubiquitous after LDIR. Very limited subset of genes has been shown to be consistently up-regulated by LDIR, including *CDKN1A*. Further research on the biological effects and mechanisms induced by IR in human cells demonstrated that the molecular and cellular processes, including transcriptional alterations, activated by LDIR are often related to protective responses and, sometimes, hormesis. Following LDIR, some distinct responses were observed, these included bystander effects, and adaptive responses. Changes in gene expression, not only at the level of mRNA, but also miRNA, have been found to crucially underlie these effects having implications for radiation protection purposes.

## 1. Introduction

The exposure of all human beings to low doses of natural and anthropogenic ionizing radiation (IR) every day is basically unavoidable. The use of IR in research, industry, homeland security, and contemporary medicine is continuously growing increasing the potential for human exposures. Some estimates suggest that an annual per capita exposure of the U.S. population to medical IR increased almost six fold between 1982 and 2006, being approximately equal to natural background IR [1]. However, the biological effects of low dose ionizing radiation (LDIR) exposures are still not adequately understood.

It is well accepted that one of the major problems in radiation research is how to extrapolate the plethora of published data on normal tissue damage and cancer risk assessment from high dose IR (HDIR) exposures to LDIR range (generally, less than 0.1 Gy). On the one hand, the latest “Biological Effects of Ionizing Radiation” (BEIR VII) report posited that the available experimental data is in accord with a “linear, no-threshold” (LNT) hypothesis [2]. This hypothesis states that even the smallest doses of IR exposures could potentially increase the cancer risk. On the other hand, the French Academy of Sciences report underscores the growing evidence for non-linearity in biological effects of LDIR, in essence, contradicting the LNT hypothesis [3]. The non-targeted effects of IR, such as radioadaptive responses (RAR), radiation-induced bystander effects (RIBE) and LDIR hypersensitivity, add to the uncertainties of assessing the biological effects of LDIR. There are indications that there may be no detectable risks, or there might even be some potential beneficial hormetic effects, at least for some experimental models and settings, associated with certain LDIR exposures [4]. A clarification of the shape of the curve for dose-response effects within the LDIR range would result in more scientifically sound estimates of LDIR risks; and better inform public policymakers on the potential outcomes of LDIR exposures setting justified and validated standards for the sake of optimal IR protection with potentially enormous socio-economic consequences.

Although there are many published reports available, the fundamental biological processes/pathways triggered by exposures to LDIR in human cells are still debatable, inconsistent and not fully conclusive. A number of epidemiological studies are available for LDIR exposures below 0.1 Gy on stochastic effects such as cancer incidence and effects on heredity [5,6]. Some data suggest that the use of computed tomography (CT) scans may almost triple the risk of leukemia in children exposed to about 0.05 Gy of LDIR [7]. Moreover, it was reported that 0.06 Gy of LDIR exposures might increase the risk of brain cancer threefold [7]. The inherent limitations of epidemiological studies requiring very large cohorts of people to be studied necessitates the basic biology studies to understand the mechanisms occurring in human cells following LDIR exposures.

The totality of available data in radiation biology has convincingly proven that the global changes in gene expression are one of the key constituents of the biological responses to IR exposures. These published reports help to understand and elucidate both short- and long-term effects of LDIR. Several lines of evidence show that LDIR effects are generally lower than expected from HDIR exposures. The gene expression alterations as well as other processes triggered by IR exposures, such as DNA damage signaling, DNA repair, apoptosis, cell cycle checkpoint activation, and cell transformation might differ both qualitatively and quantitatively following LDIR and HDIR exposures. Importantly, most data support this conclusion making the LNT hypothesis questionable in the LDIR range. However, we still have much to learn in low-dose radiobiology, especially concerning the LDIR damage induction, its propagation and how both the genetic and epigenetic background control those processes.

Numerous genome-wide DNA microarray studies were carried out to examine the effects of both HDIR and LDIR in human cells thus far. The aim of our present review is both to illustrate what is known about how different types of human cells respond to LDIR exposures at the global transcriptional level, and to summarize the current state of knowledge regarding gene expression changes underlying LDIR-specific radioresponses, such as RAR and RIBE.

## 2. Gene Expression Changes in Human Cells Following Low Doses of Ionizing Radiation Exposure

### 2.1. The Transcriptional Responses of Human Blood Cells to LDIR Exposure

Perhaps the earliest study to profile the transcriptional response of human cells to LDIR exposures using cDNA microarray was published in 2002 [8]. In this report, the overexpression of *CDKN1A* and *GADD45A* was found to be dose-dependent, linear for doses of IR exposures higher than 2 cGy and up to 0.5 Gy, with no evidence of a threshold for induction in a human myeloid tumor cell line (ML-1) [8].

In one of the earliest pioneering attempts to compare the global gene expression alterations between LDIR and HDIR exposures, the human lymphoblastoid AHH-1 cells were treated either with 5 cGy LDIR; or, alternatively, with 0.2, 0.5, 2.0 and 10 Gy of HDIR [9]. The transcriptional signatures were analyzed 4 h post-IR. LDIR triggered the induction of a subset of 25 genes, such as *MAPK8IP2*, *GPR124*, *BMPR2*, and *AGGF1*, and the repression of 18 genes. Some of the genes showed dose-dependency in their mRNA levels (generally, within 2–3-fold over sham exposed reference cultures) within the dose range 0.05–10 Gy, among them *IER5*, *XPC*, and *TP53I3* [9]. This study identified potential biomarkers of radiation injury within a broad dose range. In later study, the NimbleGene 12 × 135K microarray corresponding to 45,033 genes/transcripts was used to analyze the gene expression changes in human lymphoblastoid AHH-1 cells cultured for 6 and 20 h following 0.1 Gy of LDIR X-ray exposures [10]. The study revealed 760 induced genes and 1222 repressed genes at 6 h after LDIR whereas there were 463 up-regulated genes and 753 down-regulated genes at 20 h following LDIR. Both early and late response shared 92 differentially expressed genes in common [10]. Among the key genes examined were *GADD45A*, *GJA1*, and *CDKN2A*, suggesting that LDIR exposures elicit profound changes in gene expression signatures in AHH-1 cells potentially affecting the overall cellular radioresponse.

The transcriptional responses of peripheral blood mononucleated cell (PBMCs) of 19 apparently healthy adult individuals exposed to LDIR spanning from 0.2 mSv to 4.9 cSv over a period of 11 to 13 years, as a result of the Chernobyl Nuclear Power Plant accident, were examined by cDNA cytokine and receptor microarrays [11]. The distinct gene expression patterns were revealed in individuals exposed to LDIR greater than 1 cSv. In more than half of volunteers, the differentially expressed genes common to all doses were TGF receptor, *CD40*, and *EB13* [11]. This study underscored the variability in gene expression signatures both among individuals and across doses within the LDIR range.

DNA microarrays were used to examine the gene expression profiles in human lymphocytes collected from four healthy volunteers after 0.1 Gy LDIR; or, alternatively, 0.25 or 0.5 Gy HDIR [12]. RNA was harvested 48 h post-IR exposures; then RNA samples were collected for gene expression analysis. For 0.1, 0.25, and 0.5 Gy, the numbers of differentially expressed genes were 86, 130 and 142, respectively. The genes such as *MAPK10*, *ATF6*, and *CYP4X1* were modulated by LDIR exposures, whereas *RAD51L1*, *DUSP16*, *REV3L*, *RAD50*, and *DCLRE1A* were modulated by HDIR exposures as reported in a study [12]. The authors found a set of 34 “consensus” IR-responsive genes common for all three doses; *SERPINB2* was consistently induced, whereas *DDX49*, *STK25*, *CREB3L2*, and *XAB2* were repressed. Importantly, the Gene Ontology analysis indicated that DNA repair and stress response, cell growth and cell differentiation, general metabolism and transcription regulation were among the biological processes/pathways mostly affected by LDIR and modestly HDIR exposures [12].

The differences in gene expression in distinct IR-exposed human lymphocyte subsets using DNA microarray technology were examined and reported [13]. Blood cells from five healthy volunteers were treated with either 0.05 Gy of LDIR or 0.5 Gy of HDIR. CD^4+^, CD^8+^, and CD^56+^ cells were purified, and total RNA isolated either 3 or 24 h post-IR. HDIR resulted in the induction of the expression of *CDKN1A*, *GADD45*, *DDB2*, *BAX*, and *PCNA* 24 h post-treatment [13]. Importantly, the magnitude of radioresponse regarding the numbers of differentially expressed genes varied significantly between distinct cell types. Early response to LDIR triggered the repression of genes that was 10 times greater for CD^4+^ cells than for all other cell types examined [13]. These downregulated genes were implicated, for the most part, in oxidative phosphorylation and protein biosynthesis. Therefore, several biological processes/pathways could be responsive to LDIR in human lymphocytes, especially in CD^4+^ cells.

Another group recently investigated the whole human blood response to both 0.05 Gy LDIR and 1 Gy HDIR exposures [14]. Cytokine and chemokine signaling pathways, immune-related and inflammation processes were specifically enriched among the genes, such as *CCR4*, *GNG11* and *PF4* differentially expressed at 0.05 Gy. In marked contrast, HDIR-responsive genes mostly belong to p53 signaling pathway, DDR and apoptosis (*AEN*, *CDKN1A* and *DDB2*) [14]. Therefore, the LDIR and HDIR exposures elicit distinct biological processes in whole human blood cells. LDIR triggers the activation of the immune response whereas HDIR results in “classical” radiation stress response at transcriptional levels.

Human PBMCs transcriptional response to LDIR as related to apoptosis was elucidated in detail [15]. The cells were exposed to various LDIR doses ranging from 2 to 10 cGy. Samples for gene expression analysis were taken at 4, 24, 48, 72 h, and up to a week post-LDIR. LDIR was found to induce early repression of the *BAX* gene in human PBMCs but these changes were restored to near control levels after 168 h [15]. Generally, the expression of the *BCL2* anti-apoptotic gene was elevated, and LDIR-induced changes were transient and disappeared within a week.

The gene expression modulation after LDIR exposures was recently examined in human CD^4+^ lymphocytes by another group [16]. To this end, whole blood samples from five healthy volunteers were irradiated in six doses between 0.5 and 50 cGy, and gene expression changes were analyzed at four different post-LDIR times. The results showed that the magnitude of gene overexpression was two-fold stronger than repression and the number of differentially expressed genes did not correlate with a dose, even at the lowest dose of 0.5 cGy. There were seven gene expression clusters with distinct dose dependence signatures. Many of the p53 signaling pathway and DDR genes increased their expression with the escalating of the dose, starting from LDIR as low as 2.5 cGy. The LDIR differentially expressed genes showing no clear dose-response changes were identified to belong to ATP metabolic process, chromatin organization, and cellular respiration with alterations elicited at the lowest LDIR exposures, such as 0.5 cGy [16].

Yet another group irradiated PBMCs from 18 healthy human volunteers with 0.1–1 Gy of X-rays [17]. In a separate subset of experiments, PBMCs from one donor were irradiated with 1 cGy; 2.5 cGy; and 5 cGy and the gene expression profiles were analyzed at 24 and 48 h post-LDIR. Importantly, the lymphocyte subsets CD^4+^ or CD^8+^ cells were examined separately. The same studies were carried out in five patients exposed to LDIR either with CT scans (up to 4.3 cGy) or by administering (F-18)-fluoro-2-deoxy-d-glucose (F-18 FDG, 0.6 cGy) [17]. Exposure of PBMCs to 10 cGy LDIR resulted in gene expression profile distinct from that in mock-treated cells. Interestingly, the transcriptional signatures were alike at 24 and 48 h post-IR exposures, suggesting the transient nature of gene expression alterations. No statistically significant gene expression changes were observed below 5 cGy, implying either the limits of sensitivity of the experimental approach or the existence of a threshold for induction of LDIR-responsive genes in this study [17].

A separate set of studies was performed in human lymphoblastoid (HL) cells from two unrelated individuals [18]. These cells were exposed to LDIR spanning the range of 1–10 cGy. Then, the transcriptional profiling was carried out, and bioinformatics approaches were used to identify the differentially expressed genes responsive to LDIR. Subsets of approximately 80 genes were shared in common after LDIR in HL from both donors [18]. Many of these genes were implicated in maintenance of homeostasis, such as molecule transport and membrane signaling, and were involved in diverse signal transduction pathways. However, the majority of LDIR-responsive genes showed no dose-dependence in their level of expression across 1–10 cGy [18]. Interestingly, some genes were apparently modulated even at doses below 1 cGy. It was found that TP53, FOS and MYC were at the center of the signaling pathways triggered in HL cells by LDIR exposures. Importantly, some constituents of the LDIR response are broadly conserved across cell types and tissues in humans, independent of other confounding factors.

Very limited data are available concerning the effects of LDIR delivered chronically with low dose rate in peripheral blood mononuclear cells. In one study, a cohort of 28 professionals was exposed to a cumulative dose of 1.9 cGy [19]. They identified 256 differentially expressed genes. Importantly, the most overrepresented biological processes/pathways were belonging to mitochondrial electron transport, NADH dehydrogenase function, and DNA packaging [19]. In human ML-1 leukemia cell line, reducing the dose rate of IR exposures over three orders of magnitude still resulted in an overexpression of the key TP53-regulated genes such as *CDKN1A*, *GADD45A*, and *HDM2* in a dose range spanning between 2 cGy of LDIR and 0.5 Gy of HDIR [20]. However, with the decrease of the rate of IR exposure the magnitude of induction of *CDKN1A* and *GADD45A* was diminishing, as it was the case for many apoptosis-related genes examined in the study. On the other hand, the expression of *HDM2* was dose rate-independent, and the same pattern was seen for a substantial number of cell cycle-related genes [20]. The gene expression alterations not only imply distinct mechanisms and molecular processes triggered either by LDIR or HDIR exposures, but also a unique propensity of different pathways/processes to be sensitive to dose rate of IR exposures.

The human inter-individual variability in gene expression, especially as it relates to LDIR exposures, still remains largely unexplored. To address it, HDIR (0.5–4 Gy) and LDIR (0.5–10 cGy) gene expression alterations with 13 DDR p53 signaling pathway genes were analyzed at 2 and 24 h post-IR exposures [21] in a human blood. Importantly, HDIR response curves were approximated with a polynomial fit whereas LDIR gene expression changes fit with a linear correlation. The *DDB2*, *FDXR* were among the most upregulated genes after HDIR; and *CCNG1*, *FDXR*, and *DDB2* constitute the key signature for 10 cGy LDIR exposure [21]. These genes could potentially serve as good LDIR biomarkers being reproducible, sensitive, and proportional in their expression with the dose. However, interpersonal variability for other genes was remarkably noticeable.

The effects of even lower doses of LDIR on gene expression in human cells were assessed before. For example, the gene expression profiles obtained in lymphocytes derived from radiation workers accumulating the total absorbed dose spanning from 0.07 to 3.9 cSv were examined [22]. They identified 78 genes, among them 21 induced and 57 repressed genes, implicated in DNA repair (*RAD52*, *LIG3*, *ERCC5*, *XPA*), stress response (*DUSP22*, *GSTP1*, *PPP2R5A*), and cell cycle proliferation/regulation (*TGFB2*, *IL16*, *RHOA*) [22]. Importantly, this study suggest that gene expression changes could be reliably detected in radiation workers chronically exposed to very LDIR reinforcing the need to strictly adhere to the As Low As Reasonably Achievable (ALARA) principle for radiation health protection.

Exposure to LDIR from medical imaging has become a public health concern, but whether it triggers significant biological responses still remains unclear. Cardiac computed tomographic angiography (CTA) is a routine medical procedure for many patients suffering from heart diseases; however, CTA was reported to be associated with induction of DNA damage. The goal of one recent study was to elucidate gene expression changes stemming from CTA. The median LDIR to the blood was estimated to be about 3 cSv, ranging from 2 to 5 cSv. The data from whole genome sequencing identified changes in the expression of 39 transcription factors implicated in the regulation of cell cycle, apoptosis, and DNA repair [23]. Genes involved in regulating apoptosis and DNA repair were significantly altered post-LDIR, including *XRCC4* (3.0-fold on average), *DDB2* (1.9-fold), and *BAX* (1.6-fold) [23]. Importantly, patients exposed to >7.5 mSv of LDIR from cardiac CTA had evidence of DNA damage induction, which was associated with apoptosis and upregulation of genes involved in DNA repair and apoptosis. As well as CTA, yet another important imaging technique, namely single-photon emission computed tomography myocardial perfusion imaging (SPECT MPI) remains a major source of LDIR exposures for cardiac patients. To examine the biological effects of LDIR from SPECT MPI, the activation of the DNA damage response (DDR) was studied with the gene expression profiling at a single-cell level. The majority of 63 patients enrolled in this particular study revealed either decreased or no change in expression levels in DDR genes at both 24 and 48 h post-SPECT [24]. Importantly, within a broad cohort of patients there was a small subset of human subjects showing significant overexpression of DDR genes, suggesting that some people could potentially be more sensitive to LDIR exposures. Therefore, the results of this study warrant that all necessary precautions are taken to reduce LDIR exposures to both the patients and operators further upholding the ALARA principle.

The dose- and time-dependent gene expression alterations in human lymphocytes after exposures to both LDIR and HDIR were studied with the aim of identifying biomarker genes suitable for biodosimetry in the LDIR range [25]. Blood from six healthy volunteers was exposed to either 2 cGy, or 0.1 Gy LDIR. Four different doses above LDIR range were used (between 0.5 and 4 Gy) at 6, 24 and 48 h post-HDIR [25]. DNA microarray analysis determined nine genes with which LDIR exposures could be accurately predicted with a sensitivity of 95.6%. Importantly, within the HDIR range, the gene expression changes increased with escalating dose and time after exposure. Gene Ontology studies identified apoptosis, DNA repair, and nucleosome assembly among the biological processes/pathways mostly affected by IR exposures [25]. The transcriptional alterations in human lymphocytes triggered by LDIR as low as 2 cGy are well-defined and reliably reproducible making this experimental system a good candidate for further biodosimetry studies.

How LDIR exposures affect some of the epigenetic profiles of human lymphoblast cells have begun to be addressed. The expression signatures of microRNAs isolated from IR-exposed IM9 human cells following either 0.05 Gy LDIR or 10 Gy HDIR at 8 h post-treatment were examined with microarrays, and subsets of data were confirmed with qRT-PCR [26]. Mir-20 and mir-21 were found to be repressed by LDIR, whereas mir-197 and mir-199a were potently induced more than four-fold by HDIR [26]. These specific miRNA species could potentially be used as new biomarkers of IR-exposed human immune cells.

Overall, the data on gene expression changes triggered by LDIR exposures in human blood cells reveal the high degree of complexity of transcriptional changes. These alterations are often transient, and may not depend on dose of IR within LD range. Many “consensus” gene expression signatures identified after LDIR are yet to be verified and confirmed in larger and independent studies. What emerges is that both genetic and epigenetic background may affect the response of human blood cells to LDIR, and that such response is almost invariantly distinct from that observed following HDIR exposures.

### 2.2. The Global Gene Expression Changes in Human Skin Cells in Response to Low Doses of Ionizing Radiation Exposures

Skin is the organ in the body that is unavoidably heavily exposed to various environmental stressors, including IR. In one of the earliest published studies, cultured primary human keratinocytes harvested from adult normal skin were exposed to either 1 cGy of LDIR or 2 Gy of HDIR [27]. DNA microarrays covering about 10,500 transcripts/probes were used to examine the dynamics of gene expression alterations between 3 and 72 h post-IR. The authors identified about 140 LDIR-responsive genes, most of which were differentially expressed at 48 h [27]. Therefore, LDIR was shown to trigger specific transcriptional responses in human keratinocytes distinct from those following HDIR exposures.

At about the same time a decade ago, another group reported the global gene expression alterations elicited in normal human skin fibroblasts either by 2 cGy of LDIR or 4 Gy of HDIR [28]. Both quantitative and qualitative differences were found between LDIR- and HDIR-triggered signatures. LDIR profiles reflected the induction of DDR, signal transduction, and cell-cell signaling, whereas HDIR exposures resulted in apoptosis. Importantly, *GPR51*, *ANLN*, *KRT15*, and *GRAP2* were found to be specific to LDIR, but not HDIR in this particular study [28]. The findings, in concert with those in [27], suggest that LDIR and HDIR exposures elicit distinct molecular processes/mechanisms at the level of transcription that could, in turn, underpin the differential effects of these treatments at the more integral, cellular level.

The transcriptional alterations in human skin cells exposed to either 10 cGy LDIR or 1 Gy HDIR at four time points post-exposures were examined [29]. The immune response, programmed cell death and cell survival were enriched after these treatments. *TNFRSF1B*, *ABCA1*, *BRCA1*, and *MLLT11* were identified as being differentially expressed across LDIR and HDIR at various time points, illuminating changes in the cellular response over time [29].

It was shown that LDIR (10 cGy) exposures elicits distinct temporal cellular responses in normal human dermal fibroblasts (NHDFs) [30]. LDIR induced the mRNA expression of specific collagen *COL1A1*, but repressed matrix metalloproteinase 1 (*MMP1*) at 24 h post-LDIR. MicroRNA microarray studies demonstrated that LDIR induced alterations in the expression level of specific miRNAs and that some of these deregulated miRNAs were unique to either the early or late RAR [30]. These results suggest that LDIR triggers distinct RAR depending on the time post-IR by altering specific miRNA profiles in NHDFs.

The earliest studies on the modulation of micro RNA (miRNA) taking part in the control of gene expression at the posttranscriptional level after LDIR exposures focused on just a few selected miRNA species [31]. The normal human fibroblast cells were exposed to either acute or chronic 0.1 Gy LDIR or 4 Gy of HDIR. Dose, dose rate and temporal differences in expression levels of miRNAs were analysed. The expression profiles of many miRNA changes after LDIR exposure to either chronic or acute 0.1 Gy. For example, the expression of let-7e, and the c-MYC miRNA cluster was induced following 0.1 Gy chronic dose but were repressed after 3 h of acute 0.1 Gy LDIR [31]. The miR-21 was overexpressed after all IR regimens used; importantly, its target genes *PTEN*, *SPRY2*, and *TPM1* were repressed. Therefore, LDIR exposures proved to be able to regulate the expression of at least some IR-responsive miRNA species contributing to the overall cellular response to such exposures.

Recently, the polycomb group RING finger protein, B-cell specific Moloney murine leukemia virus integration site 1 (BMI1), was identified as one of major modulators of radioresistance. It was shown that *BMI1* levels change gene expression profiles in normal human dermal fibroblasts (NHDFs) after exposure to 0.1 Gy of LDIR [32]. MicroRNA microarrays analysis demonstrated that BMI1 knockdown results in alterations in miRNA expression in response to LDIR. The predicted target genes of the altered miRNA species were identified to be functionally implicated in both positive and negative regulation of cell proliferation, cell cycle, cell growth, and programmed cell death [32]. Therefore, the altered miRNA expression signatures in NHDF controlled by *BMI1* could be partially responsible for radioresistance in these cells; however, this assumption needs to be proven directly in subsequent experimental studies.

The gene expression alterations of quiescent primary keratinocytes and fibroblasts exposed to doses of IR spanning from 10 cGy to 5 Gy were examined with DNA microarray technique [33]. Total RNA samples were collected 4 h after treatment. IR exposure changed the expression of 279 genes across both cell types. The authors observed: (i) Changes in fibroblasts but not in keratinocytes; (ii) Changes in keratinocytes but not in fibroblasts; and (iii) Changes in both. The majority of these responses were confined primarily to TP53 target genes [33]. Therefore, TP53 target gene expression alterations are responsible for the overall cellular responses to IR, especially following HDIR.

Interestingly, some proteins were shown to be modulated by doses as low as 1 cGy and 0.1 Gy of LDIR exposures in human keratinocytes [34]. Among these protein changes the authors observed the induction of HINT1 and the repression of HSP27 [34]. In future studies it would be interesting to determine if these protein changes were accompanied by mRNA expression alterations.

There is increasing awareness that conventional 2-D cell culture models may not fully represent all the necessary conditions, such as signaling interactions, much needed to understand radiation response [35]. Therefore, some research groups have recently begun to pursue studies into LDIR exposures utilizing human 3-D tissue models. Their aim is to uncover pathways regulated by LDIR to understand how human systems respond to minor alterations in their environment and prioritize biological pathways for human health/risk assessment. Using an *in-vitro* 3-D human full thickness skin model, the temporal response of dermal and epidermal layers to 10 cGy X-ray was studied using multiple “omics” platforms, including whole genome transcriptomic approaches. As a result, the bioinformatics analysis revealed several genes as significant regulators, including some transcription factors (MYC, YY1, and CREB1), kinases (PLK1, CDK2) and a protease (MMP2) [36]. Interestingly, a temporal shift in response to LDIR was observed between 24–72 h post-exposure, with an increase in tissue remodeling, DNA repair, and repression of cell proliferation. Common molecular responses to LDIR, including nitric oxide signaling, oxidative stress, and transcriptional regulation through the SP1 factor were identified. Another study focused on the gene expression changes in response to LDIR (10 cGy) in the EpiDermFT human skin model [37]. The results identified 3299 differentially expressed genes, among which there were several long noncoding RNAs. Gene Ontology analysis demonstrated that genes belonging to the “regulation of cell proliferation” category were overrepresented [37]. Moreover, both KRAS pathway and transcription targets of NF-κB signaling pathway were enriched in response to LDIR, possibly as a result of induction of pro-survival responses.

Another group exposed EpiDermFT skin plugs to either 0.1 Gy of LDIR or 1 Gy of HDIR (X-rays) and then harvested RNA samples at 5 min; 3 h; 8 h; and 24 h post-IR [38]. The global gene expression analysis showed that HDIR triggered a change in expression of a larger number of genes over the course of studies compared to LDIR. Interestingly, during the “early” response (within the first 3 h post-IR), LDIR exposures resulted in differential expression of a larger number of genes than HDIR [38]. LDIR-responsive genes were mostly associated with survival and cell-cell signaling. HDIR exposures induced genes promoting programmed cell death at 3 h post-IR [38]. LDIR response dynamics is distinct compared to HDIR. Importantly, LDIR-triggered gene expression alterations promoted pro-survival pathways in EpiDermFT skin plugs whereas HDIR elicited genes were implicated in programmed cell death pathways [38].

Microarray-based profiling of the transcriptional response of an *in-vitro* 3-D human skin tissue model to 0.1 Gy LDIR exposures was examined [39]. The data showed significant changes in gene expression in more than 1400 genes in the dermis and more than 400 genes in the epidermis suggesting high cell type-specificity of transcriptional alterations [39]. Only a few genes were shared in common between dermis and epidermis after LDIR. Gene Ontology analysis identified immune responses, cell cycle regulation, DNA repair, and hypoxia as being the most affected pathways/processes following LDIR. The study illustrates the complexity of the transcriptional profile in human skin even after LDIR, underscoring the importance of adherence to the ALARA principle both in medical imaging/diagnostics and therapy.

Until recently it was not clear whether LDIR exposures affect cellular redox homeostasis in normal human skin cells. The studies were performed in skin fibroblast HS27 cells after 0.05 Gy LDIR and 0.5 Gy HDIR exposures [40]. Interestingly, LDIR triggered only Nrf1 activation whereas HDIR induced both Nrf1 and Nrf2 activation [40], underlying the importance of the dose-response effect in potentiating LDIR effects on the activation of Nrf transcription factors involved in cellular redox events.

In human epidermal cells, the involvement of the miRNA machinery in the cellular response to both LDIR and HDIR remains to be explored. The clarification of the mechanisms of cutaneous radiosensitivity has important practical implications both in diagnostics and therapy since skin is considered to be among the most vulnerable organs in terms of exposure to IR and ranks among the most sensitive. The human keratinocytes in culture were irradiated at 1 cGy LDIR or 6 Gy HDIR; and miRNA gene expression was analyzed three hours post-IR exposures with a 700 miRNA microarray platform [41]. Importantly, proliferative keratinocytes responded to HDIR exposures with a global decrease of miRNA expression whereas differentiated non-proliferating cells exposed to the same HDIR dose displayed a global increase of miRNAs expression. About twenty miRNA species were significantly modulated after HDIR. In marked contrast, only two miRNAs were modulated following LDIR in proliferating keratinocytes [41]. Gene Ontology analysis of miRNA predicted targets identified G-protein related pathways as being potentially modulated by these IR-responding miRNAs. Therefore, the human primary keratinocytes exposed to both LDIR and HDIR expressed a miRNA signature correlated to the differentiation status of these cells. Interestingly, the results indicate that some of differentially expressed miRNAs apparently potentiated a short-term survival of HDIR-exposed keratinocytes [41].

The transcriptional responses of the normal skin of men undergoing therapeutic radiation for prostate cancer to a single exposure of LDIR embraced seven gene groups and five biological processes/pathways [42]. These included the stress response and apoptosis pathways; Akt/phosphoinositide-3-kinase pathway; the growth factor pathway; TGFB signaling, the zinc finger protein superfamily; the keratin superfamily; and the mitogen-activated protein kinase gene group [42]. Importantly, the considerable interindividual variability was detected in gene expression signatures after LDIR exposures. Therefore, the human skin cells sense the LDIR injury, and trigger an inflammatory response with DNA remodeling, with major constituents serving to ensure the pro-survival radioresponse.

The genomic studies on the temporal dynamics of the transcriptional radioresponse of human skin after an acute exposure of 10 cGy LDIR over 24 h post-IR were performed and reported before [43]. The authors identified 19 IR-responsive gene groups and seven biological processes/pathways. Importantly, the differentially expressed genes showed only transient transcriptional changes in the healthy full-thickness human skin, returning to baseline expression levels by 24 h post-LDIR [43]. Gene Ontology analysis indicated that genes involved in inflammation, tissue remodeling, and cell cycle transition were among the most affected by LDIR. However, the interpersonal variability in gene expression profiles between patients was substantial warranting inclusion of larger cohorts of volunteers/patients in such studies potentially making them more representative.

The comparative study of the global transcriptional responses of human skin after 5 cGy LDIR and 5 Gy HDIR was carried out [44]. The authors examined the dynamics of gene expression changes at 2, 8 and 30 h after IR. LDIR and HDIR responses at the level of mRNA were distinct both qualitatively and quantitatively [44]. Three groups of differentially expressed genes were identified, among them (i) dose-dependent and time-dependent genes defined as responsive to either LDIR or HDIR but not both; (ii) unique genes defined as being responsive to either LDIR or HDIR but not both and not responsive to time after IR exposures; and (iii) dose-independent IR-responsive genes. In LDIR exposed *ex vivo* irradiated human skin they observed up to twofold differences in transcriptional responses compared to HDIR-treated skin. Importantly, gene expression changes elicited by 5 cGy were transient, whereas HDIR exposures triggered persistent gene expression alterations [44]. However, these persistent changes were found to be limited for a rather limited subset of genes. Surprisingly, neither TP53 nor TGF-beta target genes were elicited after LDIR, in a marked contrast to other published data [37].

How do types of IR exposures other than X-rays and/or gamma rays affect global gene expression in 3-D human tissues? Studies on this were performed using EPI-200, a 3-D tissue model of human epidermis, after exposure to both 2.5 Gy HDIR and 0.1 Gy LDIR of low-LET(low linear energy transfer) protons [45]. The transcriptional profiles were analyzed at 4, 16 and 24 h post-IR. Interestingly, LDIR exposures elicited recovery and tissue repair, whereas HDIR triggered terminal differentiation and loss of structural integrity. Gene Ontology studies demonstrated that TP53 dominated the HDIR response. Importantly, the transcription factor *HNF4A* was involved in LDIR responses, not only at the level of mRNA but also at total protein and phosphoprotein level [45].

The totality of published data on gene expression alterations elicited by LDIR exposures in human skin cells identified patterns inherent to different types of cells constituting skin as a whole. The global transcriptional changes are often dose-independent, and in general are quite discordant across multiple independent studies. Apparently, 3-D architecture of the human skin has to be preserved to more accurately model this LDIR-triggered gene expression changes, and such recent studies using 3-D artificial skin tissue models are relevant and highly warranted for future progress in this area.

### 2.3. The Transcriptional Responses of Human Embryonic and Mesenchymal Stem Cells to Low Doses of Ionizing Radiation Exposures

The earliest stages of human development are among the most sensitive to stress exposures, especially genotoxic stresses; and human embryonic stem cells (hESCs) represent the appropriate *in vitro* model to study the biological effects of IR exposures in this regard. However, how hESCs respond to LDIR exposures, particularly within the clinical diagnostic relevant dose range, have only recently begun to be addressed. Recently, we analyzed the gene expression changes in a panel of various hESC lines after LDIR of 0.01; 0.05; 0.1 Gy exposures; and, as a reference, relative to HDIR of 1 Gy [46]. The dynamics of transcriptional alterations of a well-established IR-responsive set of genes, such as *CDKN1A*, *GADD45A*, *etc*. at “early” 2 h and “late” 16 h post-IR suggest that stress gene radioresponses in hESCs are highly temporal- and cell line-dependent. However, we found no statistically significant evidence for a linear dose-response relationship within 0.01–0.1 Gy dose range of LDIR exposures [46].

Later, we studied the dynamics of the whole genome transcriptional alterations in different hESC lines to both LDIR and, as a reference, HDIR (1 Gy). We showed that even doses as low as 0.05 Gy could elicit significant transient changes in expression in a small subset of genes in all hESCs lines that we examined [47]. Gene expression profiles of hESCs exposed to both LDIR and HDIR were highly time-, dose-, and cell line-dependent. Utilizing a highly statistically robust bioinformatics approach we were able to identify 50 genes that constitute a “consensus” gene expression signature as an essential part of an early response to HDIR across all lines of hESC examined. We found substantial global differences in biological pathways and processes affected by either LDIR or HDIR in different lines of hESCs. This suggests that the molecular mechanisms underlying the radioresponses of hESC may fundamentally differ depending on doses of IR; however, more extensive studies with inclusion of additional lines of hESC are definitely warranted to generalize our initial findings.

How LET of IR exposures affect gene expression in human stem cells remains largely unanswered. To investigate the differential effects of low-LET X-rays and high-LET ^56^Fe ions on human mesenchymal stem cells (hMSC) a transcriptomic profiling was performed [48]. Gene Ontology studies found that receptor signaling and cytoskeleton were particularly enriched for 0.1 Gy LDIR of X-rays. On the other hand, cell cycle regulation and DNA/RNA metabolism were overrepresented for 1 Gy HDIR of X-rays and ^56^Fe ions. Importantly, more profound effects were observed after ^56^Fe ion IR exposures: DNA replication and DNA binding/transferase activity were affected more severely by 1 Gy HDIR of ^56^Fe ions than by 1 Gy HDIR of X-rays [48]. Therefore, the relative health risks associated with high-LET radiation, such as space IR, might be higher in hMSCs than those after low-LET X-rays in a comparable dose.

DNA microarray analysis was used to elucidate the differentially expressed genes and biological pathways involved in radioresponse of immortalized hMSCs exposed to either 1 cGy or 5 cGy LDIR; or, alternatively, to 0.2 Gy or 1 Gy of HDIR [49]. The gene expression signatures were analyzed at 1, 4, 12 and 48 h post-treatment. Importantly, more than six thousands of genes were shown to be differentially modulated in their expression level among 10,800 genes included in the study [49]. The dose-dependent IR-responsive genes were implicated in the regulation of transcription, proteolysis, peptidolysis, signal transduction, and general metabolism. The genes with distinct temporal responses were divided into two big subsets: (i) genes with an early induction following by repression in expression levels were associated with stress response, apoptosis, cell adhesion, and immune response; and (ii) the genes that were initially downregulated and then induced were implicated in DNA repair, DNA replication, mitosis, RNA splicing, and translation initiation [49]. Interestingly, no apparent linear relationship was found between the levels of gene expression and dose and/or time post-IR for IR-responsive genes in this study suggesting distinct and complex molecular processes triggered by either LDIR or HDIR in hMSCs.

The scarcity of available data regarding the transcriptional changes elicited by LDIR in human stem cells still imposes major limitations on our general understanding of the LDIR responses. Much more detailed and comprehensive studies wait to be performed to advance the knowledge associated with potential links between human stem cell exposures to LDIR, gene expression changes, and IR-induced carcinogenesis.

### 2.4. The Global Gene Expression Alterations in Other Types of Human Cells in Response to Low Doses of Ionizing Radiation Exposures

Among the types of human cells profiled for their transcriptional response to LDIR are umbilical vein endothelial cells (HUVECs). In one study, the cultured HUVECs were exposed to IR spanning from LDIR 2 cGy up to HDIR 2 Gy, and then allowed to recover for 4 h before RNA was harvested for DNA microarray analysis [50]. The data revealed 4134 differentially expressed transcripts. Importantly, only a few genes were modulated in a dose-dependent manner; and even the lowest dose of LDIR of 2 cGy resulted in reproducible and robust transcriptional changes in HUVECs. The consensus gene expression signature included 111 genes modulated across all doses of IR exposures [50]. Gene Ontology analysis indicated that the structural constituent of ribosomes, coagulation and peroxidase activity were among the most affected by IR exposures in HUVECs. *PRCKB1*, *CDK6*, and *TIE* were consistently repressed by all doses of IR exposures [50].

Protracted LDIR exposures were shown to cause premature senescence in at least some types of human cells, including HUVECs [51]. Importantly, the dose rate could be a critical factor in induction of such premature senescence, since this effect was observed only in HUVEC following three and six weeks of chronic LDIR regime with 4.1 mGy/h, but not 1.4 mGy/h dose rate. DNA microarray technique was instrumental in uncovering *IGFBP5* as a key driver of premature senescence in this experimental model. Studies like this call for more detailed analysis of the link between gene expression alterations and LDIR-induced senescence in other types of human cells.

Another important factor that needs to be considered in a context of LDIR is the dose rate, and how it influences the transcriptional responses in human cells exposed to LDIR. Recently, a direct comparison of genome-wide gene expression alterations in primary human prostate fibroblast cells exposed to either acute LDIR (10 cGy) or HDIR 2 Gy; and longer-term chronic LDIR (1.0–2.45 cGy delivered over 24 h) was performed [52]. Expression profiling studies found significant differential regulation of 396 genes; however, there were no measureable changes following acute 10 cGy LDIR. Importantly, there were 106 genes shared in common among IR-exposed cells given HDIR compared to those given chronic LDIR. The majority of these genes were repressed and related to chromosomal movement in M-phase, cell cycle, cell survival, DNA replication, recombination and DNA repair [52]. Interestingly, the activation of transcriptional regulators like TP53, RB1 and CDKN2A was observed. The results indicated that even chronic LDIR could elicit the changes in gene expression comparable to those of HDIR 100 times as intense suggesting the key importance of dose rate in triggering global transcriptional alterations in these human cells. This study contradicts the generally held point of view that the effects/risks following protracted low-dose rate exposures are likely less than those for a comparable dose of an acute short-term exposures [53].

The genome wide transcriptional responses of TPC-1 human cell culture of papillary thyroid carcinoma exposed to various LDIR and HDIR doses, such as 6.25 cGy; 0.5 Gy; and 4 Gy, were studied using Affymetrix microarrays [54]. A substantial overlap was identified for 4 Gy HDIR in both RET/PTC-positive systems but no common genes at 6.25 cGy [54]. The response of microRNAs in TPC-1 cells after IR exposures revealed a signature of IR-responsive microRNAs. Therefore, LDIR exposures might have a minor, anti-proliferative effect on human thyroid cells.

Gene expression profiles of human thyroid epithelial Htori-3 cells exposed to 10 cGy LDIR from iron ion HZE (high atomic number and energy) particles were examined using DNA microarray technology [55]. They found 215 differentially expressed genes two hours post-exposure, among them *IGFBP3*, *GDF15*, *B4GALT1*, *FOLR1*, and others. Importantly, LDIR exposures upregulated many genes belonging to the cytokine/chemokine gene cluster, that is, *IL6*, *IL8*, *IL11*, *IL24*, *CXCL1*, *CXCL2*, and *TGFB2* [55]. The results confirmed that LDIR stemming from HZE-particle exposures can trigger the acute immune/inflammatory responses in human thyroid epithelial cells.

One of the key parameters affecting the biological effects of IR exposures is the LET. However, how the energy deposition patterns produced by different types of IR exposures influences the transcriptional response is far from being understood. Human bronchial epithelial cells (HBECs) were exposed to either gamma-rays, or two different particle (^28^Si and ^56^Fe) IR with distinct LET [56]. The radiation species was the main segregating factor in gene expression profiles among samples, whereas the time after IR exposures was less important. Interestingly, the IR dose was not a significant factor for categorization of IR response [56]. Some genes implicated in p53 signaling pathway, such as *BTG2*, *CDKN1A*, and *TRIM22* were indiscriminately responding to IR of different LET [56]. Different radiation species triggered distinct gene expression changes among HBECs. Importantly, genes involved in cell cycle regulation and cell death were enriched for all radiation types, but particle radiation specifically triggered pro-inflammatory pathway signaling [56]. The gene profile signature comprising a set of 73 genes predicting with high accuracy the type of IR exposures was identified. This study suggests that LET of IR exposures, and not the dose of IR *per se*, might be the most important issue in determining the IR-induced gene expression changes, at least in HBECs.

As can be seen from those data, LDIR exposures trigger highly cell-type specific transcriptional response across distinct human cells. Taken into account that more than 200 types of cells constitute the human body, this finding poses major challenge to our comprehensive understanding of the human response to LDIR.

### 2.5. Gene Expression Changes in Human Cells in Radioadaptive Response to LDIR

One of the commonly observed effects of LDIR exposure in human cells is the so-called radioadaptive response (RAR). It represents a mechanism whereby LDIR exposure (priming dose) elicits radioresistance to a higher dose (challenging dose) thus substantially reducing its harmful effects. It has been suggested that LDIR leads to changes in the gene expression profiles, thereby protecting the cells from the detrimental effects of a challenging dose exposure. IR-induced DNA damage is known to be repaired through various DNA repair pathways depending upon the type of lesion in human cells. Recently, several studies have been carried out to investigate the involvement of both non-homologous end joining (NHEJ) and base excision repair (BER) genes and proteins in human PBMC under RAR settings [57,58]. BER pathway repairs IR-induced single-strand breaks, base damage, and abasic sites in both nuclear and mitochondrial DNA whereas NHEJ is involved in fixing DNA double stranded breaks (DSBs) in human cells. Human PBMCs were purified and exposed to priming LDIR of 0.1 Gy followed 4 h later with a challenging HDIR of 2.0 Gy. The expression profiles of BER and NHEJ genes and proteins were examined 30 min and up to four hours after the challenging dose using qRT-PCR reaction and western blotting, respectively. BER genes such as *APE1*, *FEN1* and *LIG1* showed significant up-regulation at mRNA level as well as at the protein level for APE1, MBD4, OGG1, FEN1 and LIG1 in primed cells [58]. NHEJ genes *XRCC5*, *XRCC6*, *NHEJ1* and *LIG4* were statistically significantly overexpressed at four hours post-irradiation both at the transcript and protein levels [57]. Overexpression of some BER and NHEJ genes and proteins in primed resting PBMCs underlies the active involvement of both BER and NHEJ pathways in human RAR. However, the rather limited number of participants enrolled in these studies warrants further studies to validate these findings in larger prospective cohorts of human subjects. Also, the phenomenon of RAR at the transcriptional level was recently examined in the AG01522 human fibroblasts first exposed to 5 cGy priming dose and then followed by 2 Gy challenging dose of X-rays [59]. Both mRNA and microRNA microarray analyses were performed and the differentially expressed genes were identified using bioinformatics approaches. LDIR exposures elicited cellular alert responses to protect against subsequent challenging HDIR damage. Therefore, RAR facilitated and accelerated the cellular repair processes under these conditions. Importantly, the stress-induced p53 signaling pathway played a crucial role in mediating DDR at the early time points after HDIR. Additional microRNA analyses demonstrated that intercellular signaling transduction and cell communication processes were overrepresented among other pathways/processes following LDIR. These data underscore the importance of global gene expression alterations in human cells of different types in manifestation of RAR after IR exposures.

Gene expression profiles of DDR genes in PBMCs were recently examined to assess their RAR [60]. Blood samples were collected from 25 healthy male donors and exposed to both LDIR and HDIR at doses between 0.1 and 2 Gy [60]. Gene expression changes were studied at 1 and 5 h post-IR. Three different priming doses of LDIR (0.1, 0.3, and 0.6 Gy) followed by a challenging dose of 2 Gy HDIR after 4 h were used. The level of expression of *ATM*, *ATR*, *GADD45A*, *CDKN1A*, *TP53*, *CDK2*, *HDM2*, and *CCNE* was studied using RT-qPCR. The data showed a significant dose-dependent induction of *CDKN1A* and *GADD45A* genes up to 1 Gy at 5 h post-IR. RAR was observed only with *TP53*, *CDK2*, and *CCNE* [60].

The gene expression changes in the transcriptional signatures emphasize the importance of global transcriptional response in manifestation of RAR, and add to understanding of the mechanism of RAR in different types of susceptible human cells.

### 2.6. Global Gene Expression Alterations in Human Cells in Radiation-Induced Bystander Effect

The gene expression changes in human cells within a context of the radiation-induced bystander effect (RIBE) are only recently begun to be explored systematically. For example, human F11 fibroblasts were exposed to LDIR of alpha-particles and transcriptional responses were assessed with a genome-wide microarray analysis [61]. The bystander cell recipients of growth medium from alpha particle irradiated (0.1, 0.5 and 2 Gy) immortalized human F11 fibroblasts were studied. The irradiated conditioned medium (ICM) was harvested and transferred from directly IR-exposed cells to bystander fibroblasts 2 h after IR. Fibroblasts irradiated with 0.1 Gy LDIR showed downregulation of 26 genes 4 h post-IR [61]. Importantly, no differentially expressed genes were identified in bystander fibroblasts at any of the time points examined in this study. The p53 stress signaling, proteasome, ribosome, and protein export pathways/processes were overrepresented in directly IR hit cells [61]. Therefore, the RIBE was accompanied with only minor transcriptional responses under these experimental conditions.

The bystander cells may initiate an apoptotic response if exposed to LDIR in 5 cGy compared to directly irradiated cells [62]. The gene expression profile for the bystander cell cultures was distinct and more complex in comparison to the directly IR-exposed cultures. The 5 cGy dose elicited a more robust apoptosic gene expression signature compared to the higher 0.5 Gy dose [62]. The differentially expressed genes were sometimes expressed not at 1 h but only at 24h post-treatment. The bystander data highlighted the induction of *TP53*, *BAX*, *BCL2*, *CASP2* and *CASP6*; however, both *CASP3* and *CASP7* were repressed after 5 cGy and 0.5 Gy exposures [62]. The results indicate that the programmed cell death could be initiated in the induction of pro-apoptotic and initiator genes but may not proceed to cell execution due to down-regulation of effector genes.

## 3. Conclusions

Responses of human cells to LDIR exposures have been a subject of extensive research spanning the last several decades. These studies have shown that the molecular-, cellular-, and tissue-level responses are different after LDIR compared to those following HDIR exposure. LDIR exposures within 0–10 cGy trigger a dual effect on human cellular DNA. One effect is a relatively low probability of DNA damage per energy deposition event that increases in proportion to the escalating LDIR. The other effect would be a protective response against DNA damage from many, mostly endogenous, sources. These will definitely depend on cell type, genotype and/or epigenotype. Such an adaptive protection would cause DNA repair and, in context of the whole body, immune system activation. Within LDIR range reduction of damage from endogenous sources could be equal or even higher compared to IR-induced molecular damage. Therefore, it is not surprising that the molecular and cellular processes elicited by LDIR are often related to protective and/or pro-survival responses. On the other hand, HDIR are often associated with cell killing, and gross perturbation of tissue homeostasis. Therefore, the underlying mechanisms of action could be unique for LDIR exposures.

The studies into LDIR exposures in human cells involving high-throughput genomic techniques such as DNA microarrays demonstrated that even LDIR exposures below 1 cGy could potentially trigger measurable changes in global gene expression. However, these alterations are often not lasting, and may not depend on dose of IR within LD range. DNA microarray datasets suggest that LDIR responses are highly genotype, cell type, and tissue-dependent, with a remarkable degree of variability both between individuals and different cell types.

Apparently, both genetic and epigenetic background affects the response of human cells to LDIR exposures. Unfortunately, many of “consensus” gene expression signatures identified after LDIR were not proven in independent studies, and many LDIR-responsive genes vary among published reports. The recent expansion of usage of proteomics, metabolomics, lipidomics, and other novel techniques necessitates the integration of global gene expression data obtained from human cells after LDIR exposures to the other “omics” approaches run in parallel with transcriptomics studies. However, the low dose radiation biology field is still in its infancy regarding the implementation of such an integration endeavors. The advantage of large-scale data integration may lie in gaining fundamentally new knowledge about the mechanisms of LDIR effects in humans which may not be obvious if only limited numbers of specific “omics” techniques are taken into account. On the other hand, distinct “omics” approaches may have different sensitivity and specificity concerning LDIR effects, and such data integration must first overcome those technical hurdles in order to make sets of big data derived from “omics” directly comparable, and analysis based on these underlying data meaningful. It is only recently that the studies to identify links between genetic, reflected on transcriptional level, and epigenetic patterns of IR responses, such as miRNAome responses, in human cells are starting to be carried out [63]. Hopefully, these data integration approaches in a context of low dose radiation biology research prove fruitful in subsequent studies, such as in [36]. In addition, human stem cell transcriptional responses to LDIR have only recently begun to be examined, and these data will help to explore the potential link between human stem cell gene expression changes, and IR-induced carcinogenesis.

The link between global changes in gene expression and cellular radiosensitivity has been a focus of research in many studies in the past, including [8,38]. What emerges from these reports is that in some experimental setups the ultimate cellular outcome depends on these gene expression alterations, which may not directly involve cell killing and/or apoptosis, but the transient cell cycle arrest [8]. Such transcriptional responses might be pro-survival [38,42], however there is a possibility that the surviving cells may carry risk of increased mutation load potentially contributing to carcinogenesis. However, such a cause-effect relationship needs to be tested rigorously in each particular case to prove the link between LDIR exposures and cancer risk. Unfortunately, the majority of the published studies lack the direct examination of a correlation between transcriptional responses and radiosensitivity measured by established techniques in context of LDIR responses in human cells.

Future research is to be focused, in large part, on identifying biomarkers, using high-throughput genomic technologies, such as DNA microarray and next generation sequencing (NGS), for detection of radiosensitive cohorts of people. This task will help to determine the IR-elicited risk associated with radiology tests and to allow for a patient-specific personalized treatment in clinic. Presently, an accurate estimation of the received IR dose seems not to be easy with gene expression as readout. The studies demonstrated that transcriptional signatures of individuals exposed to LDIR more than 1 cSv are significantly perturbed which makes the DNA microarray approach relevant and valid for detection of LDIR exposures in humans. One of the major limitations of the DNA microarray platform is that the analysis is based on predetermined nucleic acid sequences included in a given specific array which may or may not cover the whole human transcriptome. This shortcoming inherent to DNA microarray technique could be overcome with the application of NGS platforms, such as RNA-seq, to LDIR biology.

However, each human cell type has its own characteristic profile of gene expression alterations elicited by LDIR, distinct from that of other irradiated human tissues and non-irradiated cells. The application of genomics as a crucial part of systems biology to studies involving LDIR exposures may help to determine which biological processes/pathways/responses are significant in induction of radiation carcinogenesis.
